# Role of tumor associated macrophages in tumor angiogenesis and lymphangiogenesis

**DOI:** 10.3389/fphys.2014.00075

**Published:** 2014-03-05

**Authors:** Vladimir Riabov, Alexandru Gudima, Nan Wang, Amanda Mickley, Alexander Orekhov, Julia Kzhyshkowska

**Affiliations:** ^1^Department of Dermatology, University Medical Center and Medical Faculty Mannheim, Ruprecht-Karls University of HeidelbergMannheim, Germany; ^2^Department of Nanopathology, Institute of General Pathology and Pathophysiology, Russian Academy of Medical SciencesMoscow, Russia; ^3^Department of Innate Immunity and Tolerance, University Medical Center and Medical Faculty Mannheim, Institute of Transfusion Medicine and Immunology, Ruprecht-Karls University of HeidelbergMannheim, Germany

**Keywords:** tumor-associated macrophages, TIE2 receptor, VEGF, LYVE-1, stabilin-1, chitinase-like protein

## Abstract

Tumor angiogenesis is an essential process for supplying rapidly growing malignant tissues with essential nutrients and oxygen. An angiogenic switch allows tumor cells to survive and grow, and provides them access to vasculature resulting in metastatic disease. Monocyte-derived macrophages recruited and reprogrammed by tumor cells serve as a major source of angiogenic factors boosting the angiogenic switch. Tumor endothelium releases angiopoietin-2 and further facilitates recruitment of TIE2 receptor expressing monocytes (TEM) into tumor sites. Tumor-associated macrophages (TAM) sense hypoxia in avascular areas of tumors, and react by production of angiogenic factors such as VEGFA. VEGFA stimulates chemotaxis of endothelial cells (EC) and macrophages. In some tumors, TAM appeared to be a major source of MMP9. Elevated expression of MMP9 by TAM mediates extracellular matrix (ECM) degradation and the release of bioactive VEGFA. Other angiogenic factors released by TAM include basic fibroblast growth factor (bFGF), thymidine phosphorylase (TP), urokinase-type plasminogen activator (uPA), and adrenomedullin (ADM). The same factors used by macrophages for the induction of angiogenesis [like vascular endothelial growth factor A (VEGF-A) and MMP9] support lymphangiogenesis. TAM can express LYVE-1, one of the established markers of lymphatic endothelium. TAM support tumor lymphangiogenesis not only by secretion of pro-lymphangiogenic factors but also by trans-differentiation into lymphatic EC. New pro-angiogenic factor YKL-40 belongs to a family of mammalian chitinase-like proteins (CLP) that act as cytokines or growth factors. Human CLP family comprises YKL-40, YKL-39, and SI-CLP. Production of all three CLP in macrophages is antagonistically regulated by cytokines. It was recently established that YKL-40 induces angiogenesis *in vitro* and in animal tumor models. YKL-40-neutralizing monoclonal antibody blocks tumor angiogenesis and progression. The role of YKL-39 and SI-CLP in tumor angiogenesis and lymphangiogenesis remains to be investigated.

## Introduction

Tumor-associated macrophages (TAM) are key cells controlling tumor angiogenesis. TAM originate from circulating monocytes which are recruited to the tumor site and programmed by tumor-derived factors such as colony-stimulating factor-1 (CSF-1), vascular endothelial growth factor A (VEGF-A) and CC chemokine ligand 2 (CCL2) (Mantovani et al., 1992; Qian and Pollard, 2010). These and other factors in the tumor microenvironment shape the TAM phenotype and skew them toward tumor-supportive M2-polarized macrophages, although M1-polarized TAM with anti-tumor activity were also reported in several types of cancer (Forssell et al., 2007; Galarneau et al., 2007; Ong et al., 2012; Sica and Mantovani, 2012). Macrophage density correlates with poor prognosis in many types of human cancer. Tumor supporting functions of TAM including stimulation of tumor cell growth and the creation of favorable conditions for tumor cell intravasation into vessels and metastatic spread are well-described in animal models of breast cancer (Lin et al., 2006; Qian and Pollard, 2010). Numerous recent studies have demonstrated that TAM function as major producers of pro-angiogenic factors in malignant tumors. The angiogenic switch is an important step in cancer progression. The formation of new blood vessels is essential for fast growing tumor cells to be supplied with nutrients and oxygen. The angiogenic switch allows tumor cells to survive and provides them access to vasculature, which may result in the escape of malignant cells into circulation and onset of metastatic disease. Macrophages recruited and reprogrammed by tumor cells produce factors mediating the angiogenic switch (Huang et al., 2002; Lin et al., 2006; Du et al., 2008). The contribution of TAM to tumor angiogenesis was described in animal models of breast cancer, melanoma, prostate cancer, cervical cancer, and ovarian cancer (Huang et al., 2002; Egami et al., 2003; Giraudo et al., 2004; Lin et al., 2006; Halin et al., 2009). A positive correlation between TAM infiltration and angiogenesis was found in many human cancers including breast cancer, melanoma, pulmonary adenocarcinoma, glioma, gastric cancer, B-cell non-Hodgkin's lymphoma, mucoepidermoid carcinoma of salivary glands, and leiomyosarcoma (Leek et al., 1996; Nishie et al., 1999; Takanami et al., 1999; Vacca et al., 1999; Torisu et al., 2000; Shieh et al., 2009; Espinosa et al., 2011; Wu et al., 2012).

Clear evidence for the role of TAM in tumor angiogenesis was reported by Lin and colleagues using the polyoma virus middle T oncogene (MMTV-PyMT) spontaneous mouse model of mammary adenocarcinoma (Lin et al., 2006). Increased infiltration of the primary tumor with macrophages was associated with an angiogenic switch. In CSF-1-null mice, macrophage infiltration of the tumor site was significantly lower and accompanied by impaired development of the vasculature network. This was a direct effect of the absence of macrophages in the tumors since the restoration of macrophage numbers in the tumors of CSF-1-null mice by the transgenic expression of CSF-1, specifically in the mammary epithelium, resulted in the increase of vessel density. VEGF was depleted in the stromal cells of tumors of CSF-1 null mice suggesting that this is a significant reason of impaired angiogenesis. Another in vivo study demonstrated that human breast cancer spheroids implanted into nude mice induced more pronounced vascularization if they were infiltrated with macrophages before implantation (Huang et al., 2002). In this model, macrophages contributed significantly to VEGF release by spheroids and increased the angiogenic response. Up to date, VEGF-A is the best characterized TAM-derived cytokine involved in tumor angiogenesis.

## VEGF production and processing by TAM

The ability of TAM to accelerate vessel growth is mediated through the up-regulation and release of several pro-angiogenic factors. The tumor microenvironment polarizes macrophages toward M2 or a mixed M1/M2 phenotype, which is characterized by elevated expression of potent pro-angiogenic factors (Ly et al., 2010; Rolny et al., 2011). Re-polarization of the TAM phenotype toward M1 manifests in inhibition of pro-angiogenic activity and elevated expression of anti-angiogenic factors such as CXC-chemokine ligand 9 (CXCL9) and IFN-β (Rolny et al., 2011). VEGF-A is known as a major pro-angiogenic cytokine released by TAM. Its levels correlate with TAM density in several types of human cancer (Valkovic et al., 2002; Shieh et al., 2009). In breast cancer, TAM produce VEGF-A in hypoxic avascular areas of tumors (Lewis et al., 2000). Recently, the MHCII^low^ subset of TAM which resides in hypoxic areas of tumors was shown to be associated with a pro-angiogenic gene signature and increased VEGF-A expression (Laoui et al., 2013). The accumulation and retention of TAM in hypoxic areas of tumors seems to be specifically regulated by hypoxia-induced factor semaphorin 3A which triggers macrophage recruitment through VEGFR1 (Casazza et al., 2013). In macrophages, hypoxia induces expression of hypoxia inducible factor (HIF-1α and HIF-2α) transcription factors, the major master regulators of VEGF-A expression (Bingle et al., 2002; Burke et al., 2002; Imtiyaz et al., 2010; Staples et al., 2011). However, recent studies reported that HIF-1α and HIF-2α may play opposing roles in tumor angiogenesis (Eubank et al., 2011; Roda et al., 2012). In a mouse melanoma model, chemical stabilization of HIF-2α in TAM stimulated production of a soluble form of the VEGF receptor (sVEGFR-1) which neutralized biological activity of VEGF-A. This resulted in reduced angiogenesis and tumor growth (Roda et al., 2012). In contrast, HIF-1α expression in TAM was responsible for VEGF-A production. In agreement with this study, co-culture of breast cancer spheroids with wild type or HIF-1α knocked out macrophages revealed an indispensable role of macrophage-expressed HIF-1α in tumor angiogenesis (Werno et al., 2010). Besides hypoxia, HIF-1-controlled VEGF-A expression can be induced by several cytokines. The production of IL1β by TAM is able to induce HIF-1α expression and VEGF-A release in tumors. It was shown that IL1β stimulates HIF-1α production in several cancer cell lines even under normoxic conditions (Jung et al., 2003). Another common TAM-produced cytokine, transforming growth factor β 1 (TGFβ 1), also contributes to VEGF-A expression in mouse macrophages through HIF-1α/β - and Smad3/4-dependent mechanisms (Jeon et al., 2007). Alternatively, VEGF-A expression can be induced by tumor-released CSF-1 (M-CSF), which acts through NF-κ B activation and, in combination with CCL2, promotes pro-angiogenic functions of macrophages (Eubank et al., 2003; Wyckoff et al., 2004). Moreover, irradiation stimulates tumor cells to produce higher levels of CSF-1 resulting in the enhanced infiltration of pro-angiogenic myeloid cells into the tumor site (Rego et al., 2013; Xu et al., 2013). Upon secretion from cells, VEGF-A associates with extracellular matrix (ECM) and its soluble form can be released by enzymatic cleavage of ECM by matrix metalloproteinases (MMPs) (Lee et al., 2005). Several studies revealed that TAM can significantly contribute to this process. In a mouse model of human ovarian cancer, TAM were found to be a major source of MMP9. Furthermore, the presence of MMP9-expressing TAM positively correlated with tumor angiogenesis, tumor growth and VEGF-A levels (Huang et al., 2002). Elevated expression of MMP9 by tumor-infiltrating inflammatory cells in a mouse pancreatic cancer model mediated the release of bioactive VEGF-A from its extracellular reservoir (Bergers et al., 2000). In a similar study using a mouse glioblastoma tumor model, MMP9-producing macrophages and TIE2+ monocytes (TEM) contributed to the release of bioactive VEGF-A from its ECM-bound form. These bone marrow-derived cells were recruited into tumors by the HIF-1 target molecule CXC-chemokine ligand CXCL12 (SDF-1α) released by tumor cells (Du et al., 2008). It should be noted that targeting CXCL12 and its receptor CXCR4 in mouse glioblastoma prevented HIF-1-mediated recruitment of pro-angiogenic TAM and TEM, reduced vasculogenesis and abrogated tumor growth after irradiation (Kioi et al., 2010). Interestingly, a recent study demonstrated that in contrast to high-dose tumor irradiation, local low-dose gamma irradiation of pancreatic carcinomas induced TAM with an anti-tumor immunostimulatory phenotype. These TAM downregulated HIF-1 expression and suppressed intratumoral VEGF-A production resulting in the normalization of tumor vasculature. This study emphasizes the plasticity of the TAM phenotype and the possibility to revert their pro-angiogenic properties through dampening of VEGF-A-dependent angiogenesis (De Palma et al., 2013; Klug et al., 2013). Altogether, most of the studies demonstrate the indispensable role of TAM in the induction of tumor angiogenesis either through the direct production of VEGF-A or modulation of its accessibility in the tumor microenvironment.

Despite the undeniable role of VEGF in tumor angiogenesis, several studies revealed that other molecular factors (presumably HIF-1-induced factors) can significantly contribute to this process (Kioi et al., 2010; Chen et al., 2012).

## Other angiogenic factors released or internalized by TAM

Besides VEGF, TAM release a panel of pro-angiogenic factors which include tumor necrosis factor α (TNFα), basic fibroblast growth factor (bFGF), thymidine phosphorylase (TP), urokinase-type plasminogen activator (uPA), adrenomedullin (ADM), and semaphorin 4D (Sema4D) (Hildenbrand et al., 1995; Leek et al., 1998; Mantovani et al., 2007; Sierra et al., 2008; Chen et al., 2011). TP stimulates the migration of endothelial cells (EC), whereas uPA mediates ECM degradation and increases vascular invasion (Hotchkiss et al., 2003; Piao et al., 2005; Basire et al., 2006; Bijnsdorp et al., 2011). Macrophage-derived TP was associated with angiogenesis and reduced survival in human glioma and intestinal type gastric cancer (Yao et al., 2001; Kawahara et al., 2010). Elevated expression of TP is correlated with a poor prognosis in breast cancer and pancreatic cancer, and uPA expression is correlated with a poor prognosis in breast cancer (Takao et al., 1998; Toi et al., 1999; Harbeck et al., 2002). Macrophage-derived IL1α directly stimulated endothelial tube formation and neovascularization, as well as growth of mouse prostate tumors (Kwon et al., 2013). TAM-derived ADM was shown to induce angiogenesis and tumor growth in a mouse model of melanoma, and can potentially be implicated in human melanoma angiogenesis (Chen et al., 2011). In mouse models of breast cancer, TAM-produced Sema4D was found to be critical for tumor angiogenesis, vessel maturation, and tumor growth (Sierra et al., 2008). Sema4D induced motility of EC via the engagement of receptor plexin B1. In Sema4D KO mice, interactions between EC and pericytes (an essential process in vessel formation) were disrupted. Another macrophage-derived pro-angiogenic factor, prostaglandin E2 (PGE_2_), can be potentially involved in pathological angiogenesis induced in tumors by therapeutic interventions which stimulate apoptosis. It was shown that apoptotic cells activated an angiogenic program in human macrophages resulting in PGE_2_-mediated endothelial cell migration (Brecht et al., 2011).

Macrophages communicate with other cell types and control tissue turnover not only by the release of various factors, but also by their internalization and degradation. This clearance function is especially effective in alternatively activated macrophages (M2) (Kzhyshkowska and Krusell, 2009). Thus, the clearance of secreted protein acidic and rich in cystein (SPARC), a soluble component of ECM that inhibits angiogenesis by the modulation of expression of VEGF and MMPs (Zhang et al., 2012a), has been demonstrated by us in human alternatively activated macrophages (Kzhyshkowska et al., 2006a). Multifunctional scavenger receptor stabilin-1 is responsible for SPARC uptake and targeting for degradation in lysosomes. We and others also found in several tumor models that stabilin-1 is expressed by TAM (Schledzewski et al., 2006; Werno et al., 2010; Algars et al., 2012; David et al., 2012), leading to the hypothesis that stabilin-1-mediated clearance of SPARC can affect tumor angiogenesis. The clearance function of TAM as a mechanism of the regulation of angiogenesis is underestimated at the moment and has to be addressed experimentally.

## TAM induce angiogenic activity in tumor cells

**Figure 1:**
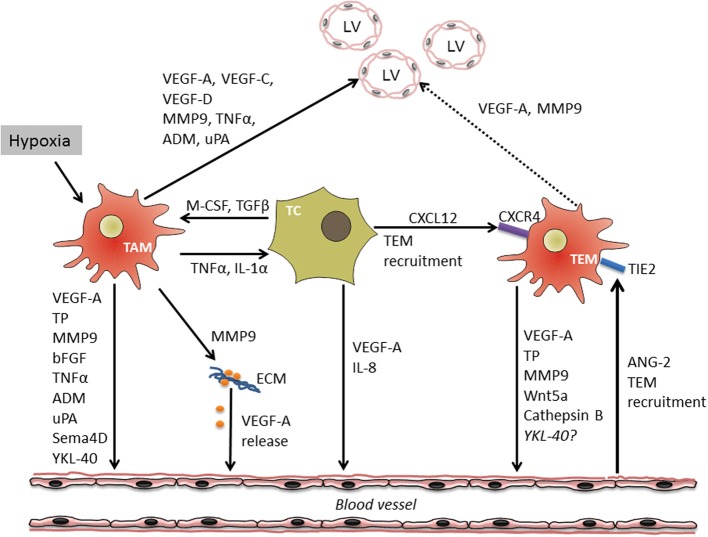
**Cross-talk between macrophages, tumor cells and endothelium during tumor angiogenesis and lymphangiogenesis**. Tumor hypoxia and cytokine crosstalk between tumor cells (TC) and TAM results in the induction of a pro-angiogenic program in both cell types followed by the release of multiple soluble factors stimulating migration and proliferation of vascular endothelial cells (VEC) either directly or through the mobilization of extracellular matrix (ECM)-bound VEGF-A. In addition, TC and VEC are able to attract specific population of TIE-2 expressing monocytes (TEM) with high angiogenic potential. TAM and TEM-derived factors as well-induce growth of tumor lymphatic vessels (LV). Through the production of factors such as VEGF-A and MMP9 influencing growth of both lymphatic and vascular EC, TAM link processes of tumor angio- and lymphangiogenesis.

The involvement of TAM in tumor angiogenesis is not limited to the self-production of angiogenic factors. TAM are able to release cytokines that indirectly contribute to tumor angiogenesis by the induction of a pro-angiogenic program in tumor cells. It was demonstrated that human monocytes and macrophages induced the production of the pro-angiogenic factors IL-8 and VEGF from melanoma and glioma cells during co-culture (Torisu et al., 2000; Hong et al., 2009). Elevated expression of IL-8 during co-culture with macrophages was also found in several lung cancer, osteogenic sarcoma and hepatoma cell lines (Chen et al., 2003). Up-regulation of IL-8 expression was presumably mediated by TAM-derived TNFα and IL1α, which stimulated NF-κ B activity in tumor cells (Yao et al., 2005). Macrophage-derived TNFα and IL-1α were necessary for the release of IL-8 and VEGF from melanoma cells (Torisu et al., 2000). *In vitro* M2-polarized monocyte-derived macrophages enhanced the angiogenic potential of human basal cell carcinoma cells through the induction of cyclooxygenase-2 (COX-2) expression resulting in the elevated release of VEGF and bFGF from tumor cells (Tjiu et al., 2009). Thus, tumor cells and recruited TAM cooperate in the tumor microenvironment to amplify the production of pro-angiogenic factors resulting in an angiogenic switch (Figure 1).

## TIE2 expressing monocytes as major inducers of tumor angiogenesis

There is a growing set of evidence that specific subpopulations of TIE2 receptor expressing monocytes (TEM) in mice and humans significantly contribute to tumor angiogenesis (Lewis et al., 2007; Matsubara et al., 2013). These monocytes/macrophages are attracted into the tumors by endothelial cell (EC)-derived cytokine angiopoietin-2 (ANG-2), which interacts with its receptor TIE2 (Huang et al., 2011). In addition, TEM express chemokine receptor CXCR4 and can be attracted into tumors by CXCL12 (Welford et al., 2011). This subpopulation of macrophages is associated with vessels and is highly angiogenic acting in a paracrine manner (De Palma et al., 2005). It is not known whether TIE2 expressing monocytes are recruited into specific types of solid tumors and whether stimulation of TIE2 expression in the conventional TAM associated with blood vessels can result into development of TEM phenotype. Blood circulating TIE2 expressing monocytes are already pre-programmed to exert pro-angiogenic activity and express elevated levels of MMP9, VEGF-A, COX-2, and Wnt5a (Coffelt et al., 2010). When stimulated by EC-derived ANG-2, TEM additionally upregulate several pro-angiogenic factors including TP and cathepsin B. Moreover, ANG-2 induces the expression of IL-10 and CCL17 by TEM, factors which suppress T-cell proliferation and promote the expansion of regulatory T-cells providing tumor cells with a way to escape from immune responses (Coffelt et al., 2011). TEM were described to cause re-growth of subcutaneous breast and lung carcinomas after local irradiation (Kozin et al., 2010). In addition, they limited the efficacy of vascular-disrupting compounds in murine mammary tumors, presumably initiating vascular repair (Welford et al., 2011). Several studies reported an important role of EC in the differentiation and functional activity of TEM. The interaction of TEM with activated EC was important for TIE2 upregulation and the establishment of a pro-angiogenic program in TEM (Mazzieri et al., 2011). A blockade of EC-derived ANG-2 in mouse MMTV-PyMT mammary cancer downregulated TIE2 expression in TEM and disrupted their association with angiogenic blood vessels. The inhibition of TIE2 expression by TEM resulted in reduced tumor angiogenesis. In another study, co-culture of primary EC with bone marrow-derived hematopoietic progenitor cells drove the differentiation of pro-angiogenic TIE2 expressing macrophages, which established tight associations with EC and supported tumor growth. The expansion of macrophage colonies was induced by EC-derived CSF-1 (He et al., 2012). Interestingly, expression profiles of TEM, resident monocytes and TIE2 expressing embryonic macrophages are related, suggesting that these myeloid populations represent developmental stages of TEM (Pucci et al., 2009). Recently, Medina and colleagues characterized a subpopulation of pro-angiogenic monocytes from human peripheral blood which were also referred to as myeloid angiogenic cells (MACs) (Medina et al., 2011). These cells had a signature of M2-polarized macrophages and expressed a panel of markers including CD163, IL-10 and macrophage scavenger receptor-1 (MSR-1). Moreover, they expressed and released a spectrum of pro-angiogenic factors such as IL-8, MMP9, and VEGF. According to the gene expression profile, MACs resembled TEM and induced endothelial tubule formation mediated mainly through IL-8 release. The role of this monocytic subpopulation in tumor angiogenesis was not described. However, their clear pro-angiogenic properties and predisposition to M2 polarization suggest a potential contribution to tumor angiogenesis once these cells are recruited to the tumor site. A summary of the complex regulation of angiogenesis by TAM is schematically presented on Figure 1.

## Effect of TAM on lymphangiogenesis

In recent years, evidence has accumulated that macrophages are not only critical regulators of angiogenesis, but also crucial participants in lymphangiogenesis, both in inflammatory settings and in tumors (Ran and Montgomery, 2012). Importantly, macrophages may simultaneously induce both angiogenesis and lymphangiogenesis by the production of VEGF-A and MMP9. These cytokines which are abundantly produced by subpopulations of TAM were shown to induce the development of both blood and lymphatic vessels. Thus, TAM-derived factors can link tumor angiogenesis and lymphangiogenesis (Scavelli et al., 2004; Coffelt et al., 2009; Gomes et al., 2013) (see Figure 1).

Macrophages can utilize two main pathways to stimulate lymphangiogenesis: either by the direct secretion of prolymphangiogenic factors or by trans-differentiation into lymphatic EC, actively taking part in the formation of lymphatic vessels (Kerjaschki, 2005). TAM can express a major marker of lymphatic vessels, LYVE-1 (lymphatic vessel endothelial hyaluronan receptor (1), both in murine and human tumors (Schledzewski et al., 2006; Zumsteg et al., 2009). It was reported that in mouse models of pancreatic insulinoma and prostate cancer F4/80^+^Lyve-1^+^ TAM directly integrated into peritumoral lymphatic vessels and presumably lost their macrophage features upon this integration (Zumsteg et al., 2009). Even though not all researchers agree with the trans-differentiation hypothesis (Gordon et al., 2010), the fact that certain macrophages secrete pro-lymphangiogenic factors in certain circumstances, such as in inflammation or tumors, is undisputed. Below we review the pro-lymphangiogenic factors secreted by tumor associated macrophages (TAM), as well as the role of TAM density in evaluating tumor lymphangiogenesis.

## Factors produced by TAM that regulate lymphangiogenesis

There are a few factors produced by TAM that are responsible for the induction of lymphangiogenesis, with VEGFR-3 and its ligands, VEGF-C and VEFG-D, thought to have a key role in it. Studies have shown that VEGF-C producing tumor cells significantly increase intratumoral lymphangiogenesis together with regional metastasis (Skobe et al., 2001a). Additionally, the inhibition of VEGFR-3 with receptor-specific antagonist antibodies was shown to suppress tumor lymphangiogenesis as well as regional and distant metastasis (Roberts et al., 2006). Schoppmann et al. found that TAM expressing VEGF-C, VEGF-D, and VEGFR-3 substantially increased tumor lymphatic microvessel density (LVD) in cervical cancer (Schoppmann et al., 2002). In another study, they showed that TAM expressing VEGF-C increased tumor lymphangiogenesis and lymphovascular invasion in breast cancer. Moreover, a positive correlation was found between VEGF-C^+^ stromal cells and VEGF-C^+^ tumor cells (Schoppmann et al., 2006). Similar findings were obtained in a study of Lewis lung carcinoma cells in which M2 macrophages displayed the ability to induce VEGF-C expression in tumor cells (Zhang et al., 2012b). By depletion of VEGFR-3^+^ TAM with clodronate liposomes, Yang et al. were able to obtain a considerable reduction (>80%) in the secretion of VEGF-C and VEGF-D in the tumor mass and also a significant reduction in LVD (Yang et al., 2011). Recently, the critical role of VEGF-C producing TAM in lymphangiogenesis and tumor dissemination was found in mantle cell lymphoma (Song et al., 2013). The description of VEGF-C expressing TAM and their prolymphangiogenic influence can also be found in a variety of other studies (Jeon et al., 2008; Moussai et al., 2011; Ding et al., 2012; Werchau et al., 2012; Wu et al., 2012).

Although VEGF-C, VEGF-D, and VEGFR-3 are mainly regarded as prolymphangiogenic factors, there are studies suggesting their role in angiogenesis. The mature forms of VEGF-C and VEFG-D possess the ability to bind to VEGFR-2, a receptor associated with angiogenesis (Joukov et al., 1997; Stacker et al., 1999; Lohela et al., 2009). Moreover, VEGF-C was shown to promote angiogenesis in vivo and to promote angiogenesis, in addition to lymphangiogenesis, in melanoma models (Cao et al., 1998; Skobe et al., 2001b). VEGFR-3 is also associated with the promotion of angiogenesis. In a study on mouse angiogenesis models, the blockage of VEGFR-3 with monoclonal antibodies was found to decrease sprouting, vascular density and endothelial cell proliferation. Additionally a blockage of VEGFR-3 in combination with the blockage of VEGFR-2 resulted in additive inhibition of angiogenesis and tumor growth (Tammela et al., 2008).

VEGF-A, a classical pro-angiogenic factor expressed by TAM, was found to be involved also in lymphangiogenesis. Apart from indirectly stimulating lymphangiogenesis by recruiting macrophages (Cursiefen et al., 2004), VEGF-A has been shown to induce proliferation and migration of VEGFR-2 expressing lymphatic endothelial cells (LEC) *in vitro* (Hong et al., 2004) and to induce sentinel lymph node lymphangiogenesis in a skin cancer model (Hirakawa et al., 2005). In a fibrosarcoma model, VEGF-A displayed the ability to induce peritumoral lymphangiogenesis as well as contribute to lymphatic metastasis (Bjorndahl et al., 2005a). Anti-VEGF-A therapy proved to be an effective way to reduce both blood and lymphatic vascular densities in a breast cancer model. Moreover, it decreased the VEGFR-3 expression levels in LEC and reduced the incidence of regional and distant metastasis (Bjorndahl et al., 2005b; Whitehurst et al., 2007).

In addition to the expression of direct inducers of lymphangiogenesis, TAM regulate lymphangioegensis, also indirectly, by the production of enzymes, such as MMP, plasmin and urokinase plasminogen activator (uPA), that regulate matrix remodeling and growth factor activation (Allavena et al., 2008). Matrix remodeling and growth factor activation are very important processes, both in angiogenesis and lymphangiogenesis. MMP-2 and MMP-9 have been shown to have a role in governing the formation of lymphatic vessels. MMP-2 facilitates LEC migration through collagen fibers, which is otherwise affected by physical matrix constraints. Inhibition or downregulation of MMP-2 and MMP-9 reduces lymphangiogenesis, the invasive ability and tube-forming properties of LEC (Nakamura et al., 2004; Detry et al., 2012) that supports the idea that MMP-2 and MMP-9-mediated lymphangiogenesis contribute their ability to promote several types of tumors. Along with MMP-1 and MMP-2, uPA was shown to have a role in lymphatic progression of oral tongue squamous cell carcinoma (Zhang et al., 2011a). Plasmin has been reported to activate the lymphangiogenic growth factors VEGF-C and VEGF-D. Proteolytic processing of these growth factors by plasmin greatly enhanced their affinity to VEGFR-3 and binding to both VEGFR-2 and VEGFR-3 (McColl et al., 2003).

Numerous other pro-lymphangiogenic factors have been identified in recent years. Among them are ADM, angiopoietin 1 and 2 (Ang-1, Ang-2), COX-2, endothelin-1, fibroblast growth factor-2 (FGF-2), growth hormone, heparanase, hepatocyte growth factor (HGF), insulin-like growth factors 1 and 2 (IGF-1, IFG-1), platelet-derived growth factor-BB (PDGF-BB) and tumor necrosis factor alpha (TNF-α) (Duong et al., 2012; Ran and Montgomery, 2012). Although these factors have been linked to lymphangiogenesis, the evidence that TAM are expressing them during carcinogenesis as part of their lymphangiogenic activity is still insufficient. Future studies will shed light on the role of TAM in the complex control of tumor lymphangiogenesis by the production of cocktails of regulators of lymphangiogenesis.

## The role of TAM density in tumor tissue and its association with lymphangiogenesis

The idea that TAM regulate tumor lymphangiogenesis is strongly supported by a significant correlation between the density of TAM and LVD in tumor tissues. In human cervical cancer, VEGF-C expressing TAM were found to correlate with increased LVD in peritumoral stroma. All TAM producing VEGF-C were also expressing VEGF-D and VEGFR-3 and were distinguished from other cells by CD68, CD14, CD23, HLA-DR, and CD45 (Schoppmann et al., 2002). In a mouse model of breast cancer, Ito et al. demonstrated a clear correlation between the density of LYVE-1^+^ lymphatic vessels, CD68^+^ macrophage infiltration, and VEGF-C expression. By comparison of breast cancer cell lines BJMC338 and BJMC3879 having low and high metastatic potential, respectively, more aggressive tumors were found to have an increased infiltration of CD68^+^ macrophages, higher expression of VEGF-C and a higher LVD (Ito et al., 2011).

In human breast cancer, higher numbers of TAM expressing VEGF-C were associated with a higher LVD and lymph node metastasis or lymph vessel invasion (LVI) (Ding et al., 2012). Similar results were found in other types of tumors as well. A study on ciliary body melanoma showed a significant correlation between LYVE-1^+^ and D2-40^+^ intraocular lymphatic vessels, higher CD68^+^ macrophage infiltration rate and an increased mortality rate (Heindl et al., 2010). Another study on melanoma revealed a correlation between CD68^+^ macrophages and D2-40^+^ lymphatic vessel invasion, but showed no association with clinical outcomes (Storr et al., 2012). In human skin squamous cell carcinoma, increased LYVE-1^+^ LVD was found to be associated with increased VEGF-C secretion by CD68^+^ and/or CD163^+^ macrophages (Moussai et al., 2011). Similar results were obtained by Werchau et al. in a study of Merkel cell carcinoma. Here, VEGF-C secreting CD68^+^ and/or CD163^+^ macrophages were also found to increase LYVE-1^+^ or D2-40^+^ LVD (Werchau et al., 2012). Unfortunately, both of these studies failed to provide a prognostic significance for these correlations. In a study of lymphangiogenesis in gastric cancer, CD68^+^ TAM were shown to be associated with a higher D2-40^+^ LVD and were closely related to serosa invasion and lymph node metastasis (Wu et al., 2012). In pancreatic cancer, VEGF-C expressing M2-polarized macrophages had an association with increased LVD density and incidence of tumor cells in regional lymph nodes (Kurahara et al., 2013).

Positive correlations between the number of TAM, LVD and tumor progression was also demonstrated for lung and esophageal cancers (Ohta et al., 2002; Kurahara et al., 2011; Zhang et al., 2011b). Not all studies were able to show a correlation between TAM, LVD and tumor progression, and some explanation to this phenomenon can be found in a recent review (Ran and Montgomery, 2012). Moreover, our examination of the TAM phenotype in pancreatic insulinoma and melanoma mouse models demonstrated that LYVE-1 can be expressed not only by lymphatic vessels, but also by TAM themselves (Schledzewski et al., 2006). Subtraction of CD68+LYVE+ macrophages has to be done for precise quantification of lymphatic vessels in tumors without counting LYVE-1+ macrophages. LYVE-1 is not the only marker expressed by both TAM and the microvascular cells in tumors. Stabilin-1 is also abundantly expressed on TAM and non-continuous endothelium and probably on lymphatic vessels (Kzhyshkowska et al., 2006b; Martens et al., 2006; Karikoski et al., 2009; Kzhyshkowska, 2010). The available information about the role of TAM in tumor lymphangiogenesis is summarized in Table 1, and a solid body of data indicate that TAM are able to support tumor lymphangiogenesis by direct and indirect effects on EC using a broad spectrum of growth factors, cytokines and enzymes overlapping with pro-angiogenic factors.

**Table 1:** **Role of TAM in tumor lymphangiogenesis and tumor progression**.

**Model**	**TAM phenotype**	**Action/correlation**	**Method**	**References**
Cervical cancer	CD68, CD14, CD23, HLA-DR, and CD45	Express VEGF-C,VEGF-D, VEGFR-3 Increased LVD in peritumoral stroma	IHC, IF, Confocal microscopy	Schoppmann et al., 2002
Lung adenocarcinoma	CD68, CD206	Increased LVD, increased lymph node metastasis rate, poor prognosis	IHC, IF, Confocal microscopy	Zhang et al., 2011b
Esophageal carcinoma	CD68	Increased microvessel density	IHC	Ohta et al., 2002
Pancreatic cancer	CD68, CD163, CD204	Increased LVD in cases with high number of CD163/CD204 TAM Increased lymph node metastasis rate in cases with high number of CD163/ CD204 TAM	IHC	Kurahara et al., 2011, 2013
Gastric cancer	CD68	Higher LVD Increased lymph node metastasis rate	IHC	Wu et al., 2012
Merkel cell carcinoma	CD68 and/or CD163	VEGF-C expression Increased LVD	IHC, IF	Werchau et al., 2012
Skin squamous cell carcinoma	CD68 and/or CD163	VEGF-C expression Increased LVD	IHC, IF	Moussai et al., 2011
Melanoma	CD68	Increased LVD	IHC	Storr et al., 2012
Ciliary body melanoma	CD68	Increased LVD Increased mortality rate	IHC	Heindl et al., 2010
Mouse model of breast cancer	CD68	Increased LVD and VEGF-C expression	IHC	Ito et al., 2011

## Chitinase-like proteins as new regulators of tumor growth and vascularization

**Figure 2:**
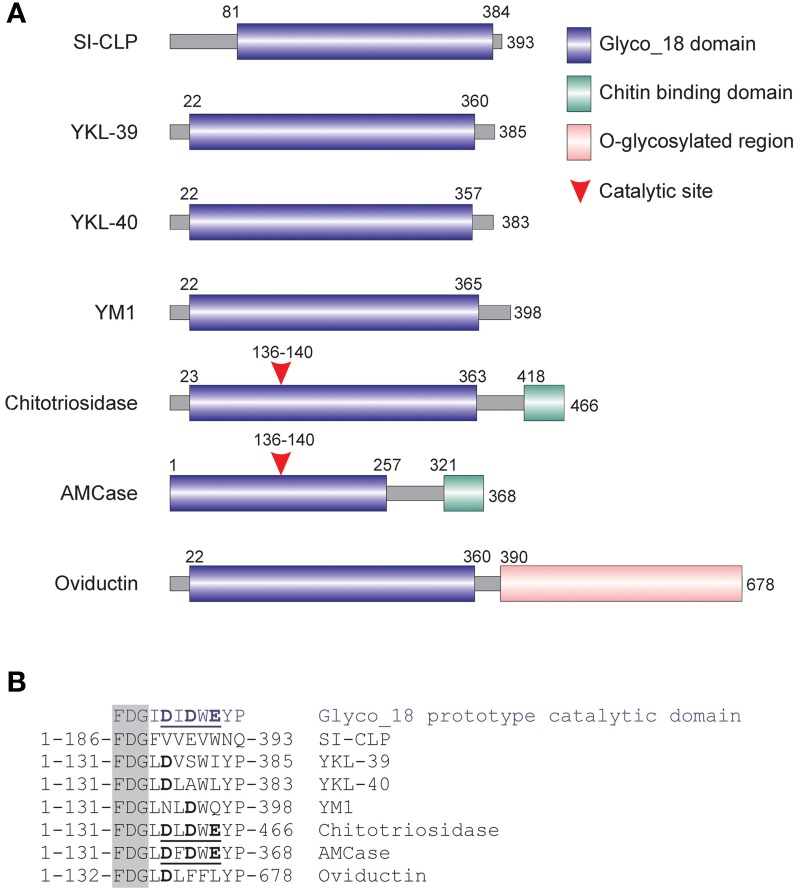
**Mammalian chitinase-like proteins (CLP) belong to the family of Glyc_18-containig proteins**. **(A)** Schematic presentation of mammalian Glyco_18 domain containing proteins. **(B)** Critical amino acid in catalytic sites. The characteristic FDG sequence preceding the catalytic motif is shown in the shadowed box. Catalytic amino acids are shown in bold. Complete active catalytic motifs are underlined. This research was originally published in *Blood*. Kzhyshkowska et al. (2006c) © the American Society of Hematology.

Recently, a new potent inducer of angiogenesis, YKL40, has been reported for several types of cancer (Shao et al., 2009; Faibish et al., 2011; Francescone et al., 2011, 2013). YKL-40 belongs to the family of Glyco_18 containing proteins that comprises chitinases and chitinase-like proteins (CLPs) (Kzhyshkowska et al., 2006c, 2007). Mammalian CLPs include YKL-39, YKL-40, stabilin-1 interacting chitinase-like protein (SI-CLP) and YM1/YM2 (only in rodents) that contain a Glyco_18 domain only; they lack critical amino acids within the catalytic site (Figure 2) and therefore do not exhibit enzymatic activity (Kzhyshkowska et al., 2006c, 2007). Their biological activity is defined by specific interactions mediated by the enzymatically silent Glyco_18 domain. The Glyco_18 domain is characteristic for the evolutionary conserved chitinases, which belong to the family of 18 glycosyl hydrolases. Enzymatically active chitinases catalyze the hydrolysis of chitin, while their evolutionary conserved function in lower life forms is to provide host defense against chitin-containing organisms (Arakane and Muthukrishnan, 2010). However, at the moment we have only limited information about the biological functions of enzymatically silent CLPs that are induced during inflammation and cancer. Major cell types that express CLPs are macrophages and tumor cells. CLPs are secreted in the extracellular space and therefore can mediate cellular crass-talk. CLPs can also be found in circulation and can act both locally and systemically.

YKL-40, also called human cartilage glycoprotein-39 (HCgp-39), gp38k and Chitinase-3-like-1 (CHI3L1), is the best investigated human chitinase-like protein regarding its association with various disorders. Increased concentrations of YKL-40 in circulation are associated with disorders characterized by different types of inflammation and active tissue remodeling, including asthma, arthritis, atherosclerosis and other cardiovascular disorders (Wang et al., 2008; Kastrup, 2011; Harutyunyan et al., 2012; Jensen et al., 2013; Konradsen et al., 2013). Circulating YKL-40 can be detected in human serum or plasma using RIA based on polyclonal antibodies. Most intensively, circulating YKL-40 was investigated in tumor patients (Johansen et al., 2009; Allin et al., 2012). Elevated levels of YKL-40 were found in the circulation of patients with various solid tumors including glioma, breast cancer, colorectal cancer, ovarian cancer, metastatic renal and prostate cancer, and malignant melanoma. In breast cancer patients, high levels of serum YKL-40 are associated with a poor prognosis (Jensen et al., 2003; Johansen et al., 2003; Kim et al., 2007; Yamac et al., 2008; Shao et al., 2011). In primary breast tumors, YKL-40 protein expression was found in tumor cells and in infiltrating inflammatory cells (Roslind et al., 2008). High expression was associated with positive estrogen and progesterone receptor status and high tumor differentiation. Serum levels of YKL-40 are indicative of a poor prognosis and rapid metastatic process. For example, increased plasma concentration of YKL-40 is related to poor prognosis and shorter survival of patients with ovarian cancer, colorectal carcinoma, metastatic prostate carcinoma and melanoma. A recent study demonstrated that YKL-40 expression in anal carcinoma is correlated with a poor outcome and can predict lymph node metastases (Mistrangelo et al., 2013). Differential levels of YKL-40 may reflect differences in the biology of cancer cells themselves, as well as the activation of innate immune responses in primary tumors, in particular, the activity and functional polarization of TAM.

YKL-40 is also the best functionally investigated protein out of all CLPs. Its stimulatory effect on tumor angiogenesis was demonstrated in several studies. Porcine homolog of YKL-40, gp38k (CHI3L1), specifically induces the migration of vascular smooth muscle cells (VSMC), but not fibroblasts. Moreover, gp38k promotes the attachment and spreading of VSMC (Nishikawa and Millis, 2003). Elevated serum levels of YKL-40 are associated with a worse prognosis among various advanced human cancers. Recently YKL-40 was found to act as a strong pro-angiogenic factor in cancer. It has been found that ectopic expression of YKL-40 in MDA-MB-231 breast cancer cells and in HCT-116 colon cancer cells led to larger tumor formation with an extensive angiogenic phenotype than did control cancer cells in mice. Affinity-purified recombinant YKL-40 protein promoted vascular endothelial cell angiogenesis *in vitro*, the effects of which are similar to the activities observed using MDA-MB-231 and HCT-116 cell-conditioned medium after transfection with YKL-40. Blockade of YKL-40 using small interfering RNA (siRNA) suppressed tumor angiogenesis *in vitro* and *in vivo*. Immunohistochemical analysis of human breast cancer showed a correlation between YKL-40 expression and blood vessel density (Shao et al., 2009). Furthermore, a potential receptor for chitinase-like protein was identified for the first time in this study. YKL-40 is a heparin-binding protein and syndecans are a major source of cell surface heparin sulfate (HS). HS functions as a key mediator connecting membrane receptors with extracellular heparin-binding proteins, such as ECM protein vitronectin and angiogenic factors (FGF, VEGF) (Lambaerts et al., 2009). Shao et al. showed that YKL-40 induces a coordination of membrane-bound receptor syndecan-1 and integrin alpha*_v_*beta_3_ and activates an intracellular signaling cascade, including focal adhesion kinase and the MAP/Erk pathway (Shao et al., 2009). YKL-40 also stimulates VEGF expression in U87 glioblastoma cell line cells and synergistically with VEGF promote angiogenesis (Francescone et al., 2011). YKL-40 is also able to enhance contact of tumor and EC, to restrict vascular leakage and stabilize vascular networks (Francescone et al., 2013).

Blocking of YKL-40 activity with monoclonal antibodies demonstrated that this can be a promising therapeutic strategy for advanced tumors (Faibish et al., 2011). A mouse monoclonal anti-YKL-40 antibody (mAY) abolished YKL-40-induced activation of VEGF receptor 2 (Flk-1/KDR) and MAP-mediated intracellular signaling, and abrogated angiogenesis induced by YKL-40 conditioned medium of the glioblastoma cell line U87 with elevated levels of YKL-40 induced by γ-irradiation. Consequently, treatment of xenografted tumor mice with mAY suppressed tumor growth and angiogenesis (Faibish et al., 2011). More information about the mode of action of YKL-40 in tumors and its prognostic and therapeutic value can be found in the recent review of Shao (2013).

YKL-39 was identified as an abundantly secreted protein in primary culture of human articular chondrocytes (Halin et al., 2009). YKL-39 is currently recognized as a biomarker for the activation of chondrocytes and osteoarthritis (OA) progression in humans. YKL-39 might be an inducer of autoimmune processes related to arthritis, while antibodies against YKL-39 were found in patients with rheumatoid arthritis (RA) and OA (reviewed in Kzhyshkowska et al., 2007). For a long time, it was believed that macrophages do not secrete YKL-39. However, recently we demonstrated that the key regulatory factor of tumor progression, TGFß, strongly stimulates YKL-39 expression in macrophages *in vitro* (Gratchev et al., 2008) suggesting that YKL39 might be a biomarker for subpopulations of macrophages that underwent programming by TGFß in the tumor microenvironment. However expression of YKL39 on macrophages in vivo remains to be examined experimentally.

SI-CLP was identified by us as stabilin-1- interacting chitinase-like protein using yeast two-hybrid screening technology (Kzhyshkowska et al., 2006c). In parallel with its sorting receptor stabilin-1, expression of SI-CLP mRNA was strongly upregulated in macrophages stimulated by Th2 cytokine IL-4 and by dexamethasone. We developed a rat monoclonal antibody, 1C11, recognizing the N-terminal epitope of SI-CLP. This epitope is located upstream of the conservative Glyco_18 domain and has no similarity with sequences of other human Glyco_18 containing proteins. Using the 1C11 antibody, we demonstrated that the combination of IL-4 and dexamethasone increases SI-CLP expression in macrophages. 1C11 mAb recognized SI-CLP in the cellular fraction of bronchoalveolar lavage specimens obtained from patients with chronic inflammatory disorders of the respiratory tract and in peripheral blood leukocytes (PBLs) from these patients. SI-CLP is the only chitinase-like protein which is upregulated by glucocorticoids. However, the expression of SI-CLP in tumor cells and TAM, and role of SI-CLP in cancer remain to be investigated.

In summary, the identification of YKL-40 as a pro-angiogenic factor in animal models and *in vitro* studies opens a new field of investigation of the specific role of CLPs in regulation of tumor growth and angiogenesis. The role of YKL-39 and SI-CLP in tumor angiogenesis has not been reported up to date, however their homology with YKL-40 makes these proteins attractive candidates for the analysis of their effects on tumor angiogenesis and such studies are in progress in our laboratory. The ability of multiple macrophage-derived pro-angiogenic factors to induce growth of both lymphatic and blood vessels raises the possibility for the involvement of YKL-40 and other CLPs proteins in the tumor lymphangiogenesis. Further experimental efforts are required in order to address the role of CLPs in lymphangiogenesis.

## Conclusions and perspectives

There are no doubts today that TAM are critical controllers of both tumor angiogenesis and lymphangiogenesis. They produce soluble factors which either directly induce vessel formation or enhance production of angiogenic factors by tumor cells. TAM-mediated support of vessel growth is associated with increased tumor growth and metastasis. Thus, targeting of TAM appears to be a promising approach for tumor therapy and can be achieved on both cellular and molecular levels. First, complete systemic depletion of macrophages can be performed. However, this approach raises significant concerns since prolonged absence of these cells in organs and circulation may result in sensitization to bacterial infections, and affect functionality of the whole immune system.

Another possibility is to block the recruitment of specific pro-angiogenic TAM populations into tumor site. Analysis of published data suggests that the disruption of CXCL12-CXCR4, ANG2-TIE2, and VEGF-VEGFR axes can prevent infiltration of tumors by angiogenic TAM populations. This approach appears to be promising to inhibit the repopulation of hypoxic tumor areas by TAM after therapeutic interventions. Finally, pro-angiogenic activity of TAM is based on, but not limited by, the release of common angiogenic factors such as VEGF-A and MMP9. Recent reports reveal novel TAM-derived angiogenic factors including ADM, Sema4D, and YKL-40 in specific tumor types. Thus, it is possible that a tumor-specific microenvironment induces the expression of distinct pro-angiogenic programs in TAM depending on tumor type and affected organ. Therefore, the identification of novel TAM-derived angiogenic factors and the validation of their targeting in specific types of cancer is urgently needed. The limitation of this method is related to the fact that TAM release a cocktail of pro-angiogenic factors, and targeting of single factor can be insufficient for suppression of tumor vascularization. Macrophages are not terminally differentiated and show high level of plasticity that makes TAM attractive targets for therapeutic immunomodulation (Stout and Suttles, 2004; Gratchev et al., 2006; Stout et al., 2009). Manipulating TAM phenotype and their re-polarization from pro-angiogenic M2 into anti-angiogenic M1 cells is an advanced strategy to solve this problem. HIF-1, COX-2, and NF-kB can be considered as promising target molecules for macrophages re-polarization. Moreover, correction of macrophage polarization in combination with targeting of single soluble factors, despite being sophisticated and requiring solid experimental investment, opens new horizons for an efficient and personalized cancer therapy.

### Conflict of interest statement

The authors declare that the research was conducted in the absence of any commercial or financial relationships that could be construed as a potential conflict of interest.
